# Case report: a co-occurring case of severe *Mycoplasma pneumoniae* pneumonia and Anti-IgLON5 antibody-associated encephalitis in a pediatric patient

**DOI:** 10.3389/fmed.2024.1393540

**Published:** 2024-08-19

**Authors:** Zimao Ye, Yuequn Chen, Xin Tian

**Affiliations:** Department of Intensive Care Unit, Fifth Affiliated Hospital of Wenzhou Medical University, Lishui Municipal Central Hospital, Lishui, China

**Keywords:** *Mycoplasma pneumoniae* pneumonia, encephalitis, extracorporeal membrane oxygenation, azithromycin, pediatric neuroimmunology

## Abstract

This case report details the clinical course of a 16-year-old female student with *Mycoplasma pneumoniae* infection complicated by autoimmune encephalitis, spanning from 6 February 2022, to 12 April 2022, with a one-year follow-up. The patient presented with a two-week history of cough and fever, followed by altered consciousness and neuropsychiatric symptoms, including hyperactivity and incoherent speech. Despite normal brain MRI findings, cerebrospinal fluid (CSF) analysis confirmed *Mycoplasma pneumoniae* with titers of, and positive IgLON5 antibodies. Initial treatment included azithromycin, ceftriaxone, and acyclovir, followed by mechanical ventilation and ECMO due to respiratory failure. The antibiotic regimen was switched to intravenous omadacycline based on genetic testing results. Autoimmune encephalitis was managed with intravenous methylprednisolone, intravenous immunoglobulin (IVIG), and plasma exchange. The patient’s condition improved, and she was discharged on 12 March 2022, with normal cognitive and behavioral functions. However, she was readmitted one month later due to cognitive decline and sleep disturbances, with a Mini-Mental State Examination (MMSE) score of 20/30 and a modified Rankin Scale (mRS) score of 3. At the one-year follow-up, her MMSE score had improved to 28/30, and her mRS score was 1. This case underscores the importance of comprehensive diagnostic approaches and personalized treatment strategies in managing complex cases of mycoplasma-related infections and associated autoimmune conditions.

## 1 Introduction

*Mycoplasma pneumoniae* is a prevalent pathogen in community-acquired pneumonia that majorly affects children and young adults, (1, 2) often presenting a range of respiratory symptoms that are usually self-limiting or responsive to antimicrobials (3). In parallel, autoimmune encephalitides, like the rare Anti-IgLON5 antibody-associated encephalitis, are a diverse set of disorders marked by immune-mediated inflammation of the central nervous system, posing considerable diagnostic and therapeutic complexities (4). This paper unfolds a unique clinical confluence of severe *Mycoplasma pneumoniae* pneumonia (MPP) and Anti-IgLON5 antibody-associated encephalitis in a 16-year-old female. It underscores the crucial role of a comprehensive diagnostic evaluation and a customized therapeutic approach, emphasizing the necessity of considering an extensive differential diagnosis in pediatric patients exhibiting simultaneous respiratory and neurological symptoms, thus enriching the evolving knowledge in managing intricate pediatric neuroimmunological disorders.

## 2 Case presentation

**FIGURE 1:**
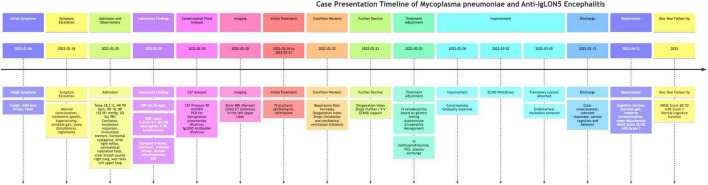
Timeline of case presentation. The timeline begins on 6 February 2022, with the onset of initial symptoms including cough, mild sore throat, and fever. By February 18, symptoms escalated to altered consciousness, incoherent speech, hyperactivity, unstable gait, sleep disturbances, and nightmares. The patient was admitted on February 20, with vital signs including a temperature of 38.2°C, heart rate of 90 beats/min, respiratory rate of 18 breaths/min, blood pressure of 125/81 mmHg, and oxygen saturation of 98%. Initial examination revealed confusion, incoherent responses, involuntary tremors, horizontal nystagmus, brisk light reflex, symmetrical nasolabial folds, clear breath sounds in the right lung, and wet rales in the left upper lung. Laboratory findings included elevated C-reactive protein, white blood cell count, and other markers indicating infection and inflammation. Initial treatment with intravenous acyclovir, azithromycin, and ceftriaxone commenced on February 20–21. On February 22, the patient’s respiratory rate increased, necessitating intubation and mechanical ventilation. Further deterioration led to ECMO support on February 23, with genetic testing confirming *Mycoplasma pneumoniae* infection. Treatment was adjusted to include intravenous omadacycline, methylprednisolone, immunoglobulin, and plasma exchange. By February 28, the patient’s condition improved, leading to ECMO withdrawal on March 2 and significant recovery by March 5, allowing for discharge on March 12. The patient experienced a relapse and was readmitted on April 12 with cognitive decline, unstable gait, impaired communication, and sleep disturbances, with cerebrospinal fluid analysis showing positive IgLON5 antibodies. At the one-year follow-up, the patient had largely regained cognitive function, maintained a stable gait, and was attending school.

The case presentation, as shown in Figure 1, spans from 6 February 2022, to 12 April 2022, with a detailed timeline of symptoms, treatments, and interventions. Additionally, a one-year follow-up is included, highlighting the patient’s recovery and ongoing symptoms. A 16-year-old female student was admitted to our facility on 20 February 2022, presenting with a two-week history of cough and fever, followed by two days of hyperactivity and incoherent speech. The initial symptoms included cough without expectoration, mild sore throat, and fever. However, two days prior to admission, the patient exhibited altered consciousness, incoherent speech, inability to engage in normal communication with family, hyperactivity, unstable gait, sleep disturbances, and was easily awakened from sleep with nightmares. We chose azithromycin for anti-infective treatment after admission because it is a first-line antibiotic effective against *Mycoplasma pneumoniae* (5), the pathogen confirmed through genetic testing (*Mycoplasma pneumoniae* Real-Time PCR Kit, Shanghai Kepeirui Biotechnology Co., Ltd). Azithromycin is known for its efficacy in treating respiratory infections, including those caused by *Mycoplasma pneumoniae*, and is also preferred due to its good safety profile in pediatric patients. In the 2 weeks before admission, the patient’s treatment regimen included other empirical antibiotics, antipyretics and cough suppressants, and maintenance medications for any chronic conditions, with adjustments made based on the patient’s response to these treatments.

Her past medical history was unremarkable with no known epilepsy or autoimmune diseases, and there was no family history of psychiatric illnesses. Upon examination, her temperature was 38.2°C, heart rate 90 beats/min, respiratory rate 18 breaths/min, blood pressure 125/81 mmHg, with an oxygen saturation of 98% at the fingertips. She stood at 161 cm tall and weighed 48 kg. The patient appeared confused, with incoherent responses, involuntary tremors in all extremities, horizontal nystagmus, equal and round pupils measuring about 3.0 mm with brisk light reflex, symmetrical nasolabial folds, and no mouth drooping. Auscultation revealed clear breath sounds in the right lung, while wet rales were noted in the left upper lung. Normal muscle tone was observed in all four limbs, with no neck stiffness, and Babinski sign was negative.

Laboratory investigations showed a C-reactive protein level of 54.16 mg/L, white blood cell count of 9.0 × 109/L with a neutrophil percentage of 85.9% and lymphocyte percentage of 9.9%. The prothrombin time was 15.7 s, D-dimer 1.25 mg/L, calcitonin 0.04 ng/ml, creatine kinase 498 U/L, lactate dehydrogenase 589 U/L, and erythrocyte sedimentation rate 120 mm/h. Respiratory viral panel and hemorrhagic fever antibodies were negative. However, the measured quantities of IgM and IgG antibodies in the patient’s sample indicate significant findings (*Mycoplasma pneumoniae* ELISA Kit, Shanghai Kepeirui Biotechnology Co., Ltd.). The IgM value of 12.90 units confirms the sample is positive for *Mycoplasma pneumoniae* IgM antibodies, suggesting an acute or recent infection. Additionally, the IgG value of greater than 300 AU/ml signifies a high level of IgG antibodies, which points to either a current or past infection with *Mycoplasma pneumoniae*. These results provide crucial insights into the patient’s immune response and infection history. Immunological profiling revealed absolute counts of helper T cells at 194/ul, cytotoxic T cells at 217/ul, with a CD4/CD8 ratio of 0.89, total T lymphocytes at 432/ul and total lymphocytes at 510/ul. Additionally, anti-myeloperoxidase antibody was at 42.8 AU/ml, lupus anticoagulant ratio was 1.31, weakly positive antinuclear antibodies with a titer of 1:100, weakly positive anti-SSA, and positive anti-Ro-52 antibodies. CSF analysis showed a pressure of 90 mmH_2_O. Using PCR kits for detection, CSF sample tested positive for *Mycoplasma pneumoniae*. As the patient experienced a fever lasting for two weeks before admission, it is crucial to consider other potential causes beyond mycoplasma-related infection, such as bacterial or viral infections. However, viral infections have been ruled out, and tests for other pathogens have returned negative results. Given the patient’s prolonged fever and the negative findings for other pathogens, the focus remains on confirming and addressing the mycoplasma-related infection as the primary cause of the symptoms.

**FIGURE 2:**
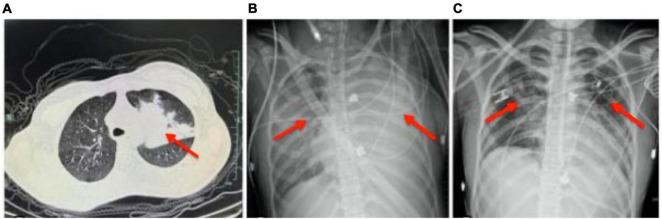
Depiction of chest imaging over the course of treatment. **(A)** Chest CT scan on February 20 showing an infection in the left upper lobe. **(B)** Bedside chest radiograph on February 23 exhibiting diffuse pulmonary infiltration, necessitating veno-venous extracorporeal membrane oxygenation (V-V ECMO) support due to a decrease in oxygenation index despite 100% oxygen support. **(C)** Bedside chest radiograph on March 5 demonstrating significant absorption of pulmonary infiltrates following tailored antimicrobial and immunotherapeutic interventions, allowing for the removal of endotracheal intubation. The depicted timeline reflects the dynamic pulmonary pathology and the impact of medical interventions, correlating with the patient’s clinical course from severe infection through to stabilization and recovery.

The brain MRI did not reveal significant abnormalities (data not shown), but a chest CT scan revealed an infection in the left upper lobe (Figure 2A). The patient was initially treated with intravenous acyclovir, azithromycin, and ceftriaxone. On February 22, her respiratory rate increased with an oxygenation index of 87, necessitating emergency endotracheal intubation and mechanical ventilation, following which she was transferred to the intensive care unit. On February 23, despite 100% oxygen support, her oxygenation index dropped to 58, leading to an emergency veno-venous extracorporeal membrane oxygenation (V-V ECMO) support. Bedside chest radiographs showed diffuse pulmonary infiltration (Figure 2B). High-throughput genetic testing of bronchoalveolar lavage fluid revealed *Mycoplasma pneumoniae* (sequence number 166191). Autoimmune encephalitis testing of blood and cerebrospinal fluid samples revealed positive IgLON5 antibodies (1:30), while antibodies against N-methyl-D-aspartate receptor (NMDAR), voltage-gated potassium channel (VGKC) complexes, Hu, Yo, and Ri were all negative. The antimicrobial regimen was switched to intravenous omadacycline. Refractory *Mycoplasma pneumoniae* pneumonia is typically treated with antibiotics such as minocycline, doxycycline, or quinolones. In this case, the child was treated with omadacycline, which is a newer tetracycline antibiotic. The choice of omadacycline was made due to its broad-spectrum activity and effectiveness against resistant strains of *Mycoplasma pneumoniae*. This treatment was part of a clinical trial designed to evaluate the efficacy and safety of omadacycline in pediatric patients with refractory *Mycoplasma pneumoniae* pneumonia. The clinical trial was conducted under the approval from the ethics committee of Fifth Affiliated Hospital of Wenzhou Medical University, with the ethics approval number No. 2022-DE-I. The use of omadacycline aimed to provide a more effective treatment option for the patient, considering the resistance patterns observed with traditional antibiotics. The change from azithromycin and ceftriaxone to omadacycline was based on the specific identification of *Mycoplasma pneumoniae*, which was resistant to traditional antibiotics. This switch was justified to address the identified pathogen effectively. Escalation of Respiratory Support: The progression from mechanical ventilation to ECMO was necessitated by the patient’s worsening respiratory status, reflecting appropriate escalation in line with her clinical needs. Introduction of Autoimmune Encephalitis Treatment: The identification of IgLON5 antibodies indicated an autoimmune component, justifying the use of methylprednisolone, IVIG, and plasma exchange. These interventions were crucial to manage the autoimmune encephalitis component of her illness. For autoimmune encephalitis, she was treated with intravenous methylprednisolone, intravenous immunoglobulin, and plasma exchange.

By February 28, the patient’s consciousness gradually improved, showing compliance with commands. On March 2, ECMO support was withdrawn; by March 5, pulmonary lesions were significantly absorbed, and endotracheal intubation was removed (Figure 2C). The patient was discharged on March 12 with clear consciousness, coherent responses, normal cognition, and behavior. One-month post-discharge, the patient was readmitted due to cognitive decline, unstable gait, impaired communication, and continued sleep disturbances with nightmares. At that time, her Mini-Mental State Examination (MMSE) score was 20/30, indicating moderate cognitive impairment, and her modified Rankin Scale (mRS) score was 3, reflecting moderate disability requiring some help but able to walk without assistance. At the one-year follow-up, her MMSE score had improved to 28/30, showing near-normal cognitive function, and her mRS score had decreased to 1, indicating no significant disability despite minor symptoms. She had largely regained cognitive function, maintained a stable gait, and was attending school, although she continued to experience nightmares, affecting her quality of life.

**FIGURE 3:**
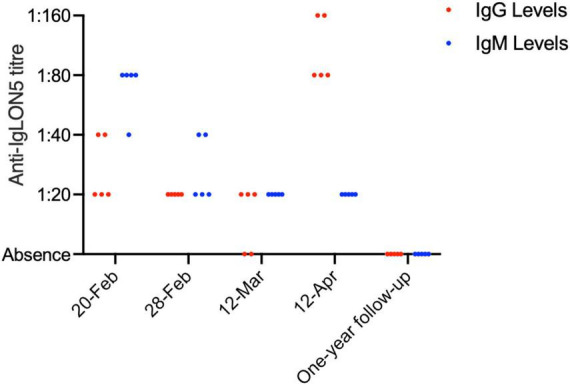
Comparative analysis of IgG and IgM antibody levels over time. The figure presents a bar chart comparing the levels of IgG (blue) and IgM (red) antibodies at various time points, indicating the progression and response to treatment in the patient over time.

On 20 February 2022, Anti-IgLON5 IgG antibody titers were recorded as 1:20 in three out of five experiments and 1:40 in two out of five experiments. Anti-IgLON5 IgM antibody titers were recorded as 1:40 in one experiment and 1:80 in four experiments. By 28 February 2022, an improvement was observed with IgG titers at 1:20 in all five experiments and IgM titers at 1:20 in three experiments and 1:40 in two experiments. Peak recovery on 12 March 2022, showed IgG titers at 1:20 in all five experiments and IgM titers at 1:20 in all five experiments. However, by 12 April 2022, during readmission, IgG titers had decreased, with three experiments showing titers at 1:80 and two experiments at 1:160, while IgM titers were recorded as 1:20 in all five experiments. At the one-year follow-up, both IgG and IgM antibody titers were considered negative (Figure 3).

## 3 Discussion

MP is a common etiological agent of community-acquired pneumonia in children and adolescents, often presenting with a persistent, severe dry cough as a principal symptom. While the majority of cases are mild, severe MPP is relatively rare, especially among immunocompetent children. Studies indicate that severe MPP accounts for approximately 0.5 to 2% of all MPP cases, (6) predominantly occurring in children (7). The mortality rate associated with severe MPP remains unclear, and 50% percent of these patients have been reported to be died (8). From the onset of MP infection to the development of respiratory failure, the average median duration is 19.5 ± 8.34 days (range, 9–31 days) (9). The exact mechanisms underlying the severity of MPP are yet to be fully elucidated; however, cellular immunity plays a significant role in disease progression. Toxic inflammatory substances produced post-infection cause cellular damage through direct interactions between host cells (10).

In the presented case of a 16-year-old healthy adolescent female with a two-week history of fever and cough, elevated MP IgM and MP IgG levels confirmed the diagnosis of MPP, and azithromycin was chosen for antimicrobial therapy. Due to rapid pulmonary progression and a low oxygenation index, high-throughput genetic testing was conducted, which confirmed severe MPP, necessitating V-V ECMO support. A literature search revealed scarce reports of V-V ECMO usage in severe MPP cases, underscoring the timeliness and crucial role of ECMO support in the successful management of our patient (11). Regarding antimicrobial therapy, macrolides like azithromycin remain the first-line treatment for MPP; however, the rapid pulmonary progression in this case might be attributed to an exaggerated inflammatory response or suboptimal antibiotic efficacy. Although glucocorticoids should be cautiously used in severe MPP due to the risk of secondary infections, their use demonstrated a positive effect in this case following rapid pulmonary progression (12). Omadacycline, a novel tetracycline antibiotic, is employed for treating community-acquired pneumonia caused by MP, (13) but there are yet no reports on its use in severe MPP within our region. Upon confirming severe MPP in this patient, azithromycin was discontinued, and omadacycline was administered to bolster antimicrobial therapy. Subsequently, the patient’s condition gradually improved, pulmonary lesions were absorbed, and ECMO support was withdrawn. The use of omadacycline in the treatment of severe MPP is still under-researched, and further studies are warranted to ascertain its efficacy in severe MPP management.

AE is a spectrum of conditions characterized by acute or subacute central nervous system inflammatory disorders triggered by autoimmune mechanisms against neuronal antigens (14). Symptoms can encompass cognitive decline, motor and behavioral abnormalities, epileptic seizures, and sleep disturbances. Etiologically, AE can be classified into infectious, post-infectious, and non-infectious types, with post-infectious and non-infectious AE accounting for about 20% of all clinically suspected encephalitis cases. Pathologically, these manifest as lymphocytic infiltrates within the brain parenchyma, forming a “sleeve-like” structure around vessels. MP infection has been validated as a trigger for autoimmune encephalitis in numerous studies, with anti N-methyl-D-aspartate receptor encephalitis being the most common subtype (15). Our patient exhibited altered consciousness, incoherent speech, and sleep disturbances. With cerebrospinal fluid (CSF) analysis ruling out infectious encephalitis, (16) further testing for non-infectious intracranial pathology was undertaken in both blood and CSF, revealing positive IgLON5 antibodies with a titer of 1:30 (Figure 3) (17). Although the titer was low, it still has diagnostic relevance as illustrated by reported cases with even lower titers.

Anti-IgLON5 disease is an autoimmune disorder that has been increasingly recognized in association with lung infections (18). The presence of positive IgLON5 antibodies in CSF is indicative of an autoimmune response triggered post-infection. It is essential to monitor patients for neurological symptoms following Mycoplasma infections, as early identification and treatment of autoimmune encephalitis can significantly improve outcomes (19, 20). Identifying autoimmune encephalitis involves recognizing clinical signs such as altered consciousness, cognitive decline, seizures, and movement disorders, in conjunction with specific antibody testing (21, 22).

To effectively diagnose autoimmune encephalitis following a Mycoplasma infection, clinicians should consider a combination of clinical evaluation, imaging, and laboratory testing (19, 23). Key steps include performing CSF analysis to detect specific autoantibodies like IgLON5, conducting brain MRI to identify any inflammatory changes, and utilizing electroencephalography (EEG) to monitor for abnormal brain activity. Additionally, it is important to be aware of the broader spectrum of post-infectious autoimmune encephalitides, which may present similarly but involve different antibodies. Early and accurate diagnosis enables the initiation of appropriate immunotherapies, such as intravenous immunoglobulin (24), corticosteroids (25), and plasma exchange, which can mitigate the autoimmune response and improve the patient’s prognosis. The normal MRI findings (data not shown) in encephalitis patients can be perplexing, particularly when clinical symptoms strongly suggest autoimmune encephalitis. Despite normal imaging results, the diagnosis cannot be ruled out because autoimmune encephalitis can sometimes present without overt structural changes visible on MRI. This emphasizes the importance of integrating multiple diagnostic modalities.

Anti-IgLON5 antibody-associated encephalitis is an exceedingly rare subtype of AE (26). As diagnostic capabilities have advanced, more cases are being reported globally, primarily in individuals aged 50 to 70 years. The disease is insidious with non-specific clinical manifestations including sleep disturbances, bulbar dysfunction, progressive supranuclear palsy-like syndrome, movement and cognitive disorders, autonomic dysfunction, peripheral nerve hyperexcitability, and bilateral vestibular abnormalities. Brain MRI scans usually reveal no characteristic abnormalities, and most patients exhibit no notable anomalies. Serum and CSF examinations might show normal or mildly elevated cell count and protein, with positive IgG IgLON5 antibodies in serum or CSF. The management of Anti-IgLON5 antibody-associated encephalitis lacks standardized guidelines, though immunotherapy is commonly employed. First-line treatment includes corticosteroids and immunoglobulins, while second-line treatment comprises plasma exchange and immunosuppressants. Early diagnosis and prompt intervention are crucial for better prognostic outcomes. In our case, the patient was administered corticosteroids, immunoglobulins, and underwent plasma exchange thrice, which led to a gradual awakening and clear cognition upon discharge.

A thorough review of both domestic and international literature revealed no reported cases of MP infection inducing Anti-IgLON5 antibody-associated encephalitis. Our patient, an adolescent female, deviates from the typical age range reported in existing literature, making a conclusive diagnosis challenging. However, from this case, we discern that when diagnosing MP infection, if azithromycin treatment proves ineffective, consideration for omadacycline and even ECMO support might be warranted. Additionally, if a patient exhibits symptoms like altered consciousness, behavioral abnormalities, or sleep disturbances, further comprehensive blood and CSF examinations, including those for AE-associated antibodies, should be pursued. This could provide a robust basis for definitive diagnosis and subsequent therapeutic interventions, enhancing the clinical management of such complex co-occurring conditions.

In this case, the reduced CD4/CD8 ratio could reflect the immune system’s response to the *Mycoplasma pneumoniae* infection and the subsequent autoimmune encephalitis (27–29). This imbalance might suggest a predominance of cytotoxic T cells over helper T cells, which can be a marker of chronic inflammation or immune activation. Additionally, a low CD4/CD8 ratio has been associated with poor prognosis in various infectious and autoimmune diseases (30), highlighting the need for careful monitoring and potentially more aggressive immunomodulatory treatments to restore immune balance and improve patient outcomes. Future studies should incorporate whole exome sequencing for the patient and his parents to ensure accurate and reliable genetic analysis, which could significantly impact the management and treatment plans for similar cases.

### 3.1 Diagnostic challenges

This case presented several diagnostic challenges due to the initial ambiguity of symptoms and their rapid progression. The overlap between infectious and neurological symptoms, such as altered consciousness and incoherent speech, made it difficult to pinpoint a single cause. Additionally, the necessity for urgent interventions due to the patient’s deteriorating respiratory status added complexity to the diagnostic process. Differentiating between an infectious etiology and autoimmune encephalitis required comprehensive testing, including serology, CSF analysis, and genetic testing. The presence of IgLON5 antibodies alongside negative results for other common autoimmune markers (NMDAR, VGKC) further complicated the diagnostic landscape.

### 3.2 Diagnostic reasoning and differential diagnosis

The diagnostic reasoning process involved a systematic assessment of symptoms and test results. Initially, the patient’s respiratory symptoms suggested an infection, justifying the use of antibiotics such as azithromycin and ceftriaxone. However, the emergence of neurological symptoms prompted further investigation into possible encephalitis. Differential diagnoses included viral encephalitis, bacterial infections, and autoimmune encephalitis. The confirmation of *Mycoplasma pneumoniae* through genetic testing and serology, combined with the detection of IgLON5 antibodies, supported a diagnosis of *Mycoplasma pneumoniae* pneumonia complicated by autoimmune encephalitis. The patient’s improvement following immunomodulatory treatments (methylprednisolone, immunoglobulin, plasma exchange) validated this diagnosis.

### 3.3 Prognostic characteristics

The patient’s prognosis was significantly influenced by the timely and aggressive intervention strategies employed. Early recognition of the condition and the use of advanced therapies, such as ECMO and switching to omadacycline, were crucial in stabilizing the patient. The patient showed marked improvement in consciousness and respiratory function following treatment, indicating a positive response. Long-term follow-up revealed substantial recovery in cognitive function and physical abilities, with the patient’s MMSE score improving to 28/30 and her mRS score reducing to 1. Despite ongoing challenges, such as persistent nightmares, the patient’s overall prognosis was favorable, underscoring the importance of multidisciplinary care and regular monitoring.

### 3.4 Strengths of the approach

The choice of azithromycin as an initial treatment was justified due to its broad-spectrum antibacterial properties and anti-inflammatory effects, making it suitable for empirical therapy. Azithromycin’s efficacy against *Mycoplasma pneumoniae* and its convenient dosing regimen enhance patient compliance. The subsequent use of omadacycline, a newer tetracycline antibiotic, was appropriate given its broad-spectrum activity and effectiveness against resistant strains of *Mycoplasma pneumoniae*, especially in the context of a clinical trial aimed at evaluating its efficacy and safety in pediatric patients.

### 3.5 Limitations of the approach

A limitation of the initial approach was the need for rapid escalation to mechanical ventilation and ECMO, suggesting that initial treatments may not have been sufficiently effective in preventing respiratory deterioration. Additionally, while azithromycin has anti-inflammatory properties, its use alone was insufficient to manage the severe autoimmune encephalitis component, necessitating the addition of immunosuppressive therapies like methylprednisolone, intravenous immunoglobulin, and plasma exchange.

## 4 Conclusion

This intricate case of a 16-year-old female presents a unique convergence of severe *Mycoplasma pneumoniae* pneumonia (MPP) and Anti-IgLON5 antibody-associated encephalitis, underscoring the diagnostic and therapeutic challenges intrinsic to such complex clinical manifestations. Comprehensive and adaptive therapeutic strategies, involving tailored antimicrobial and immunomodulatory approaches, were pivotal in navigating the patient through critical respiratory distress and nuanced neurological impairments, facilitating significant clinical amelioration. However, the persistence of certain neurological sequelae underscores the protracted nature of recovery in such intricate cases and necessitates ongoing clinical vigilance. This detailed clinical odyssey reinforces the imperative of a nuanced, multifaceted approach to diagnosis and management in cases marked by a convergence of infectious and autoimmune etiologies.

## Data availability statement

The raw data supporting the conclusions of this article will be made available by the authors, without undue reservation.

## Ethics statement

The studies involving humans were approved by the Hospital Ethics Committee of The Fifth Affiliated Hospital of Wenzhou Medical University, Lishui Central Hospital. The studies were conducted in accordance with the local legislation and institutional requirements. Written informed consent for participation in this study was provided by the participants’ legal guardians/next of kin. Written informed consent was obtained from the participant/patient(s) and/or the the participants’ legal guardians/next of kin for the publication of this case report.

## Author contributions

ZY: Data curation, Formal analysis, Methodology, Resources, Writing – original draft. YC: Formal analysis, Investigation, Methodology, Software, Writing – original draft. XT: Formal analysis, Funding acquisition, Methodology, Writing – review & editing.
